# Hypotension Prediction Index Software Compared with Standard Advanced Haemodynamic Monitoring in Patients Undergoing Major Aortic Surgery: A Retrospective Study

**DOI:** 10.3390/jcm14248791

**Published:** 2025-12-12

**Authors:** Jakub Szrama, Mariusz Gezela, Łukasz Żurański, Katarzyna Kulas, Michał Gajda, Piotr Smuszkiewicz, Paweł Sobczyński

**Affiliations:** 1 Department of Anaesthesiology, Intensive Therapy and Pain Management, Poznan University of Medical Sciences, 60-355 Poznan, Poland; 2 First Department of Anesthesiology and Intensive Therapy, Poznan University of Medical Sciences, 61-848 Poznan, Poland; mariusz.gezela@usk.poznan.pl (M.G.); lukasz.zuranski@usk.poznan.pl (Ł.Ż.); katarzyna.kulas@usk.poznan.pl (K.K.); michal.gajda@usk.poznan.pl (M.G.); pawel.sobczynski@usk.poznan.pl (P.S.)

**Keywords:** hypotension, major vascular surgery, aortic abdominal surgery, haemodynamic monitoring, Hypotension Prediction Index, Acumen IQ, FloTrac

## Abstract

**Background/Objectives:** Intraoperative hypotension (IOH) is related to the occurrence of postoperative complications and may be a frequent event during major vascular surgery. The Hypotension Prediction Index (HPI) is a technology applied to predict hypotension and enable preventive interventions. This study aimed to compare intraoperative haemodynamic stability between patients monitored with the HPI algorithm and those monitored with arterial pressure cardiac output (APCO) monitoring. **Methods:** We performed a retrospective study including 100 adult patients undergoing elective major aortic surgery between January 2023 and June 2025. Fifty patients were managed with APCO monitoring and 50 with the HPI algorithm. The primary endpoint was time-weighted average mean arterial pressure below 65 mmHg (TWA-MAP < 65 mmHg). Secondary endpoints included total hypotension time, number and duration of hypotensive episodes, and time spent with MAP > 90 and > 100 mmHg. Multiple comparison correction (Holm–Bonferroni) was applied separately for hypotension and hypertension outcomes. **Results:** The primary outcome, TWA-MAP < 65 mmHg, did not differ significantly between groups (0.22 vs. 0.26 mmHg; p=0.27). After correction for multiple comparisons, no hypotension-related outcomes reached statistical significance, although clinically relevant trends were observed: the HPI group showed 50% shorter total hypotension time (5 vs. 10 min; $p_{\mathrm{uncorrected}}=0.03$, $p_{\mathrm{adjusted}}=0.18$) and 33% shorter episode duration. In contrast, patients in the HPI group spent significantly more time with elevated MAP: 38% vs. 25% of monitored time with MAP > 90 mmHg ($p_{\mathrm{adjusted}}=0.036$) and 18% vs. 9% with MAP > 100 mmHg ($p_{\mathrm{adjusted}}=0.036$). **Conclusions:** In patients undergoing major vascular aortic surgery, HPI monitoring did not significantly reduce the burden of hypotension after accounting for multiple comparisons, though clinically meaningful trends were noted. However, HPI use was associated with significantly increased hypertensive exposure, suggesting overly aggressive correction. These findings highlight the need for careful titration of interventions when using predictive algorithms and warrant further prospective randomised studies.

## 1. Introduction

The cornerstone of anaesthetic perioperative management is to maintain stable cardiovascular function during the surgical procedure. Perioperative hypotension is quite common and is related to serious postoperative complications, including myocardial injury, kidney failure and even death [1,2,3]. Patients undergoing vascular procedures have some of the highest rates of major adverse cardiovascular and cerebrovascular events (MACCE), including perioperative myocardial infarction, stroke and death, compared with those undergoing other non-cardiac surgeries [4,5,6]. The American College of Cardiology and the American Heart Association emphasise that vascular surgery carries a substantially higher risk of perioperative cardiovascular events, with an incidence of MACCE of almost 7.7% for vascular procedures, which is higher than for general, orthopaedic or genitourinary surgeries [4,6]. Postoperative myocardial infarction following major vascular surgery is associated with early mortality rates of over 16%, while perioperative stroke, though less common, increases 30-day mortality rates and prolongs hospital stays [7,8].

Cardiovascular stability during surgery can be achieved by applying various haemodynamic protocols which take into account parameters derived from invasive arterial blood pressure and cardiac output monitors. This so-called goal-directed haemodynamic approach has proved to be beneficial in certain clinical scenarios [9,10,11,12,13,14,15]. However, perioperative goal-directed therapy (GDT) has not been shown to reduce postoperative complications or mortality, particularly in patients undergoing vascular surgery. A meta-analysis of randomised controlled trials in cardiac and vascular surgery found that, although GDT reduced complications in cardiac surgery, it did not reduce morbidity or mortality in vascular surgery patients [16]. This lack of benefit may be due to distinct co-morbidities and pathophysiology in vascular surgery populations.

A randomised controlled trial of peripheral arterial surgery also found that intraoperative GDT targeting a cardiac index above 2.5 L/min/m^2^ did not improve tissue oxygen delivery or reduce cardiovascular complications compared to standard care, regardless of anaesthetic technique [17]. Systematic reviews and consensus statements emphasise that, while GDT benefits high-risk surgical patients in general, the evidence for vascular surgery is inconsistent and does not support its routine use [16,17].

Even the most sophisticated haemodynamic protocols utilise a reactive approach, which involves making a clinical intervention to an event that already occurred. The technology involving a machine learning device developed by Edwards, named the Hypotension Prediction Index (HPI), changed the haemodynamic approach from reactive to preventive. There have been studies, literature reviews and meta-analyses which showed benefit; however, we are still lacking the “organ injury” beneficial clinical studies [18,19,20,21,22]. Furthermore, there are still doubts about using this technology in vascular and cardiac surgical patients. The aim of this study was the comparison of the frequency of hypotension in major vascular surgery according to the type of advanced haemodynamic monitoring technology applied perioperatively. We hypothesise that the Hypotension Prediction Index (HPI) software reduces intraoperative hypotension compared with the arterial pressure-derived cardiac output advanced haemodynamic monitoring.

## 2. Materials and Methods

This is a retrospective single-centre study performed in the First Department of Anaesthesiology and Intensive Care of the Medical University Hospital in Poznan, Poland, between the 1 January 2023 and 30 June 2025. The study was approved by the Bioethics Committee of the Poznan University of Medical Science (the study protocol number is 409/25, date of approval 28 May 2025). The Institutional Review Board waived the requirement for informed consent because of the retrospective design of the study.

**Figure 1:**
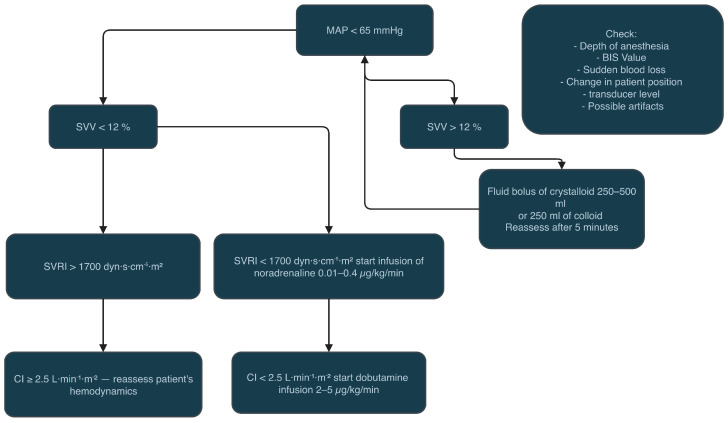
Institutional FloTrac algorithm for fluids, vasopressors and inotropes.

**Figure 2:**
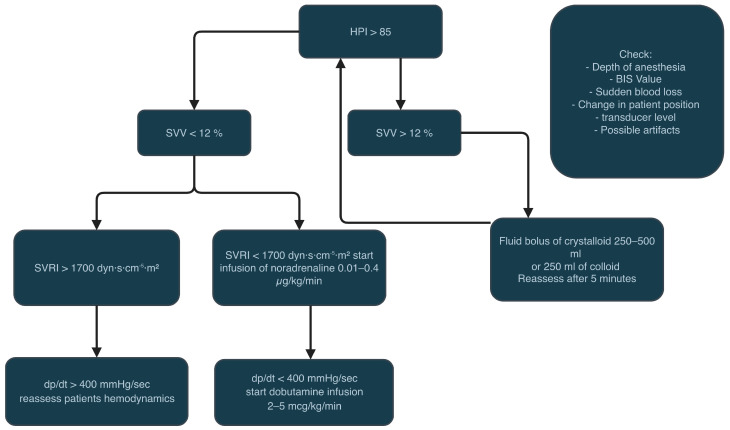
HPI-based haemodynamic treatment protocol (institutional).

We included adult patients undergoing major aortic vascular surgery under general anaesthesia who received advanced haemodynamic monitoring (HPI or arterial pressure-derived cardiac output with the FloTrac transducer). The exclusion criteria were emergency surgery, pregnancy, age under 18 years old and all the cardiac conditions making the arterial pressure cardiac output monitoring less reliable, like atrial fibrillation and severe aortic/mitral stenosis or regurgitation. All included patients underwent general anaesthesia, with the choice of the anaesthetic drugs and the type of haemodynamic monitoring made by the attending anaesthesiologist. The retrospective analysis included the comparison of patients having the haemodynamic monitoring with the use of the HPI technology—the HPI group (Acumen IQ sensor, Edwards Lifesciences, Irvine, CA, USA) and the arterial pressure-derived cardiac output monitoring—standard haemodynamic group (FloTrac sensor; Edwards Lifesciences, Irvine, CA, USA). Based on our institution protocol, all patients had an arterial line placed before induction to anaesthesia, and the haemodynamic monitoring was commenced. In the standard haemodynamic monitoring group with the FloTrac sensor, the haemodynamic data consisted of cardiac output (CO), cardiac index (CI), stroke volume (SV), stroke volume index (SVI), stroke volume variation (SVV), systemic vascular resistance (SVR) and systemic vascular resistance index (SVRI). The fluid, vasopressor and inotropic therapy in patients in the FloTrac group was managed according to our institution’s local protocol presented in Figure 1. In the haemodynamic monitoring group with the Hypotension Prediction Index and the Acumen IQ sensor, the haemodynamic data consisted of cardiac output (CO), cardiac index (CI), stroke volume (SV), stroke volume index (SVI), stroke volume variation (SVV), systemic vascular resistance (SVR), systemic vascular resistance index (SVRI), dynamic arterial elastance (${\mathrm{Ea}}_{\mathrm{dyn}}$), the ratio of pressure change in the ventricular cavity (dP/dt) and the Hypotension Prediction Index (HPI) value. As the HPI value reaches 85, the system informs about the upcoming hypotensive event, and a secondary screen appears on the monitor with the advanced haemodynamic parameters. The clinical decisions regarding the use of fluids, inotropes or vasopressors are based on the protocol presented in Figure 2.

### 2.1. Endpoints

The primary endpoint of the study was time-weighted average of mean arterial pressure (TWA-MAP) below 65 mmHg, which is the integration of duration and severity of hypotension in relation to the time of monitoring (duration of surgery/anaesthesia). TWA is calculated as follows: (rate of hypotension in millimetres of mercury below a MAP of 65 mmHg × time in minutes below MAP of 65 mmHg)/total duration of monitoring time in minutes.

$$\mathrm{TWA}\text{-}{\mathrm{MAP}}_{<65}=\frac{\mathrm{area}\;\mathrm{below}\;65\;\mathrm{mmHg}\;\left(\mathrm{mmHg}\cdot \min\right)}{\mathrm{total}\;\mathrm{monitoring}\;\mathrm{time}\;\left(\min\right)}.$$

The secondary endpoints included the total number of episodes of hypotension, total hypotension time below 65 mmHg in minutes, mean duration of the hypotensive event below 65 mmHg, number of episodes of hypotension with MAP below 50 mmHg, total hypertension time in minutes and as a percentage of total monitoring time for MAP > 90 mmHg and 100 mmHg, TWA for MAP > 90 mmHg and TWA for MAP > 100 mmHg.

### 2.2. Postoperative Outcomes

Basic data regarding postoperative complications, including mortality, need for re-operation, surgical complications, stroke, respiratory failure, circulatory failure, acute kidney injury, and myocardial injury after non-cardiac surgery (MINS), were collected. Postoperative respiratory failure was defined as a composite endpoint reflecting clinically relevant impairment of gas exchange or ventilation in the early postoperative period. It was considered present if at least one of the following criteria was met within 7 days after surgery or before ICU discharge: (1) prolonged invasive mechanical ventilation, defined as failure to extubate within 48 h after the end of surgery; (2) unplanned reintubation with initiation of invasive mechanical ventilation due to respiratory failure (hypoxaemia, hypercapnia or inability to maintain adequate ventilation) after initial extubation; or (3) unplanned initiation of non-invasive ventilation or high-flow nasal oxygen for respiratory failure, maintained for at least 6 h after extubation. Postoperative ventilatory support and airway management data were retrieved from the electronic anaesthesia and ICU records. Postoperative myocardial injury after non-cardiac surgery (MINS) was defined on the basis of high-sensitivity cardiac troponin measurements. During the study period, cardiac troponin was measured using high-sensitivity assays with assay-specific 99th percentile upper reference limits (URLs) of either 0.034 ng/mL or 0.053 ng/mL, depending on the laboratory platform in use at the time. For the purposes of this analysis, MINS was defined as at least one postoperative troponin value above the assay-specific upper reference limit (i.e., ≥URL) within 30 days after surgery, irrespective of the presence of ischaemic symptoms or ECG changes. All troponin results were obtained from the hospital laboratory information system. Postoperative circulatory failure was predefined as a composite endpoint reflecting clinically relevant cardiovascular instability in the early postoperative period. It was considered present if at least one of the following criteria was met within 48 h after surgery: (1) sustained requirement for vasopressor support, defined as continuous norepinephrine infusion ≥0.1 μg/kg/min for ≥6 h despite adequate fluid resuscitation; (2) initiation or escalation of inotropic therapy (dobutamine, milrinone or epinephrine at inotropic doses) for signs of tissue hypoperfusion; (3) need for mechanical circulatory support (intra-aortic balloon pump, ECMO or equivalent). All postoperative haemodynamic and ventilatory data were retrieved from the electronic ICU records. Full definitions of all outcomes are provided in the Supplementary Material.

### 2.3. Statistical Analysis

All the intraoperative haemodynamic parameters were exported from the HemoSphere platform and analysed using Acumen Analytics Software Version 1.0.22 (Edwards Lifesciences Corp., Irvine, CA, USA).

Categorical variables are presented as frequencies and percentages. Differences between groups were assessed using Fisher’s exact test with Monte Carlo simulation (10,000 replicates) when expected cell frequencies were less than 5, or the chi-square test otherwise. The distribution of quantitative variables was assessed with the Shapiro–Wilk test. Normally distributed variables are reported as mean ± standard deviation (SD), while non-normally distributed data are presented as median with interquartile range [IQR: 25th (Q1) to 75th (Q3) percentile]. Between-group comparisons were performed using Welch’s two-sample *t*-test for normally distributed data or the Mann–Whitney U-test for non-normally distributed data. For significant differences, mean or median differences with 95% confidence intervals (CI) are reported.

Baseline characteristics, co-morbidities, and perioperative interventions were compared using appropriate statistical tests. *p*-values for these comparisons are reported for descriptive purposes without adjustment for multiple comparisons, as these tests serve only to demonstrate group balance.

For postoperative adverse events (organ dysfunction, MINS, and other complications), no multiplicity correction was applied. These outcomes were pre-specified, clinically distinct adverse events, each representing an independent mechanism of harm. Because they do not reflect repeated testing of the same hypothesis, multiplicity adjustment is not appropriate and would inflate the Type II error, potentially obscuring true safety signals. Uncorrected *p*-values are presented for descriptive interpretation.

For primary and secondary outcomes, we applied the Holm–Bonferroni correction separately to two families of comparisons: (1) primary outcome and secondary outcomes related to hypotension (MAP < 65 mmHg; n=7 tests), and (2) secondary outcomes related to hypertension (MAP > 90 mmHg and MAP > 100 mmHg; n=6 tests). Both uncorrected ($p_{\mathrm{uncorrected}}$) and adjusted ($p_{\mathrm{adjusted}}$) *p*-values are reported. An adjusted *p*-value < 0.05 was considered statistically significant after correction for multiple comparisons.

Given the fixed sample size (n=50 per group), we performed a post-hoc power analysis to determine the minimum detectable effect (MDE) for the primary endpoint at α=0.05 and 80% power. Precision of estimates was summarised using 95% CI widths.

To interpret the non-significant result for the primary endpoint (TWA–MAP < 65 mmHg) within a clinically relevant framework, we applied the confidence–interval-based method recently proposed by De Cassai [23]. This approach quantifies how much of the 95% CI lies beyond a predefined minimal clinically important difference (MCID). As no validated MCID exists for TWA-based hypotension burden, we defined the MCID as δ=0.28 mmHg, corresponding to the minimum detectable effect (MDE) at 80% power for our study design (n=50 per group). For the observed effect $\Delta_{e}$ with CI boundaries $\left(\Delta_{L},\Delta_{U}\right)$, clinical relevance was evaluated by determining whether the CI exceeded the MCID threshold or lay entirely below it. When $\Delta_{U}<\delta$, the data were interpreted as evidence against any clinically meaningful benefit of HPI compared with conventional monitoring with arterial pressure-derived cardiac output utilising the FloTrac transducer.

Statistical analyses were performed using MedCalc^®^ Statistical Software version 20.115 (MedCalc Software Ltd., Ostend, Belgium; 2022) and R (R Foundation for Statistical Computing, Vienna, Austria; version 4.5.1 (13 June 2025)).

## 3. Results

The study included data derived from patients undergoing major aortic vascular surgery between the 1 January 2023 and 30 June 2025. We analysed data from 100 patients, with 50 having arterial pressure–derived cardiac output monitoring with the FloTrac transducer (FloTrac group) and 50 having Hypotension Prediction Index haemodynamic monitoring with the Acumen IQ transducer (HPI group). There were no statistically significant differences between the analysed groups in the basic demographic data, like age, gender, and BMI. There were no statistically significant differences in the preoperative ASA status, co-morbidities (except heart failure and coronary artery disease, which were present more often in the HPI group), and administered drugs (Table 1). The types of vascular procedures are reported in Table 2. The distribution of surgical procedure types did not differ significantly between the FloTrac and HPI groups (Fisher’s exact test, p=0.463). The most common procedures in both groups were aorto-bifemoral bifurcated grafts (FloTrac: 50%, HPI: 38%) and straight aortic grafts (FloTrac: 20%, HPI: 38%).

Similarly, we reported no differences in perioperative management, including administered fluids, blood products, and noradrenaline infusion. These results are presented in Table 3.

**Figure 3:**
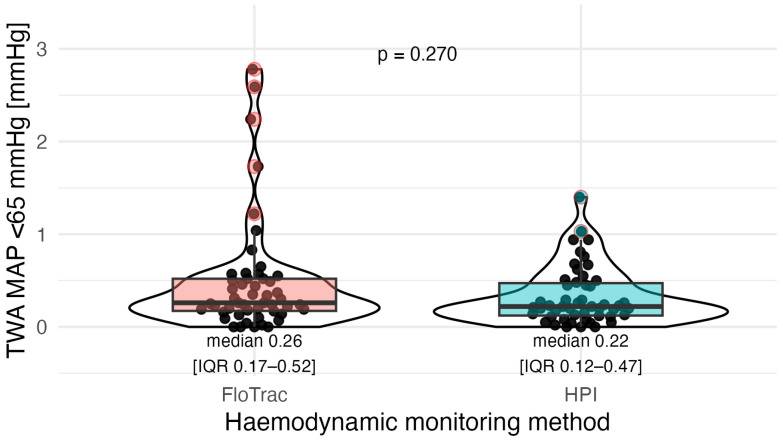
TWA MAP < 65 mmHg values in FloTrac (red) vs. HPI (blue) groups. Violin plots show distribution density with boxplots (median and IQR); outliers (beyond 1.5 × IQR) are marked with red borders. Median [IQR]: 0.26 [0.17–0.52] vs. 0.22 [0.12–0.47]; $p_{\mathrm{uncorrected}}=0.27$ (not significant).

The primary endpoint of the study, the median value of TWA-MAP < 65 mmHg, did not differ significantly between the groups (0.26 mmHg in the FloTrac group and 0.22 mmHg in the HPI group; p=0.27; Figure 3).

Using the CI–MCID framework of De Cassai [23], the Hodges–Lehmann difference for the primary endpoint was $\Delta_{e}=0.05$ mmHg, with a 95% confidence interval ranging from $\Delta_{L}=-0.04$ to $\Delta_{U}=0.15$ mmHg (CI width: 0.19 mmHg).

The minimum detectable effect (MDE) was 0.28 mmHg for 80% power and 0.32 mmHg for 90% power. The predefined minimal clinically important difference (MCID) was therefore set to δ=0.28 mmHg. The upper confidence interval boundary remained below this threshold ($\Delta_{U}<\delta$), resulting in a proportion above MCID of $P_{\delta }=0\%$.

There was also no statistically significant difference in the number of hypotensive episodes below 65 mmHg (p=0.154) or 50 mmHg (p=0.562). After Holm–Bonferroni correction for multiple comparisons within the primary outcome family (*n* = seven comparisons), none of the hypotension parameters reached statistical significance. However, patients in the HPI group showed clinically relevant trends: shorter total hypotension time (median 5 min [IQR 2–11] vs. 10 min [5–15]; median difference −5 min [95% CI −10 to −1]; $p_{\mathrm{uncorrected}}=0.03$, $p_{\mathrm{adjusted}}=0.18$; see Supplementary Figure S1) and shorter mean episode duration (median 2 min [IQR 1–3] vs. 3 min [2–3]; $p_{\mathrm{uncorrected}}=0.02$, $p_{\mathrm{adjusted}}=0.14$). These represent 50% and 33% reductions, respectively, which may be clinically meaningful despite not reaching statistical significance after correction. The data on hypotension with MAP < 65 mmHg are presented in Table 4.

Analysis of hypertension episodes revealed an unexpected finding. After Holm–Bonferroni correction for secondary outcome comparisons (*n* = 6), patients in the HPI group spent significantly more time in hypertension. Patients in the HPI group spent a median of 62 min vs. 49 min in the FloTrac group with MAP > 90 mmHg ($p_{\mathrm{uncorrected}}=0.046$; see Supplementary Figure S3a). Furthermore, episodes of hypertension with MAP > 90 mmHg constituted 38% of total monitored time in the HPI group vs. 25% in the FloTrac group ($p_{\mathrm{uncorrected}}=0.006$, $p_{\mathrm{adjusted}}=0.036$; see Supplementary Figure S3b).

The results were similar for episodes of hypertension with MAP > 100 mmHg: 31 min in the HPI group vs. 16 min in the FloTrac group ($p_{\mathrm{uncorrected}}=0.02$), representing 18% vs. 9% of total monitored time, respectively ($p_{\mathrm{uncorrected}}=0.006$, $p_{\mathrm{adjusted}}=0.036$) (see Supplementary Figures S2–S4; Table 5). These findings suggest potential overshooting of blood pressure targets with HPI-guided management.

There was no statistically significant difference in the incidence of postoperative complications between the FloTrac and HPI groups. Detailed frequencies of adverse events are shown in Table 6.

## 4. Discussion

Our retrospective study compares two modalities of advanced haemodynamic algorithms, with the APCO and FloTrac being a “reactive” approach and the HPI being a “preventive” approach, applied in patients undergoing major aortic vascular surgery. The primary outcome, TWA MAP < 65 mmHg, did not differ significantly between groups. After applying Holm–Bonferroni correction for multiple comparisons, none of the hypotension-related outcomes (MAP < 65 mmHg) reached statistical significance, although clinically relevant trends were observed: the HPI group showed 50% shorter total hypotension time and 33% shorter mean episode duration. In contrast, patients in the HPI group spent significantly more time with MAP > 90 mmHg (38% vs. 25% of monitored time, $p_{\mathrm{adjusted}}=0.036$) and MAP > 100 mmHg (18% vs. 9% of monitored time, $p_{\mathrm{adjusted}}=0.036$), suggesting more aggressive management to avoid hypotension may have resulted in increased hypertensive exposure.

### 4.1. Haemodynamic Management in Vascular Surgery

Haemodynamic management during major aortic vascular surgery is one of the most challenging tasks for the anaesthesiologist. Vascular surgery patients are especially vulnerable due to a high prevalence of co-morbidities such as hypertension, atherosclerosis, and chronic kidney disease, which increase susceptibility to hypoperfusion and ischaemic injury during hypotensive episodes. Common perioperative complications associated with major vascular surgery include myocardial injury, acute kidney injury, stroke, congestive heart failure, postoperative mechanical ventilation, and increased early mortality. These complications are strongly associated with intraoperative hypotension, particularly when mean arterial pressure (MAP) falls below 65 mmHg for prolonged periods [6,18]. It is mandatory to remember that open abdominal aortic aneurysm repair presents unavoidable haemodynamic challenges due to surgical manipulation such as aortic clamping and declamping, as well as reperfusion. Consequently, haemodynamic changes (like hypotension and hypertension) are often sudden and acute. The HPI technology does not effectively capture anaesthetic management prior to declamping or predict the timing of declamping. The key factors of anaesthetic management are rapid and optimal responses to these abrupt changes during surgical management. Due to these factors there are still doubts about the usefulness of the HPI algorithm during vascular and cardiac surgeries. The possible benefit of this technology is the reduction in hypotension during parts of surgery not related to aorta manipulation, therefore reducing the total duration of hypotension.

Despite these well-recognised challenges, the intraoperative applicability of functional haemodynamic monitors such as the HPI algorithm remains debated. Their performance is inherently limited during phases of extreme surgical manipulation, where abrupt, mechanically induced shifts in preload and afterload cannot be reliably anticipated. Nevertheless, during more stable periods of the procedure, these monitors may still enhance haemodynamic situational awareness and support more targeted fluid or vasopressor therapy. In this context, their potential benefit lies not in predicting clamp-related haemodynamic instability, but in reducing cumulative hypotension burden during non-critical phases of surgery, which may ultimately contribute to improved postoperative recovery.

There are many studies and systematic reviews showing benefits in reducing episodes of hypotension with the utilisation of the HPI algorithm [19,20,21,22]. However, the number of studies focusing on vascular surgery patients is scarce.

### 4.2. Clinical Interpretation of the Primary Endpoint

Although the primary endpoint (TWA–MAP < 65 mmHg) did not differ statistically between groups, interpretation of non-significant findings requires an assessment of clinical as well as statistical relevance. The observed Hodges–Lehmann difference between HPI and FloTrac was only 0.05 mmHg, with a 95% confidence interval (CI) from −0.04 to 0.15 mmHg. To contextualise these results, we applied the confidence-interval–based framework recently proposed by De Cassai [23], which compares the CI to a predefined minimal clinically important difference (MCID).

Because no established MCID exists for TWA-based hypotension burden, we defined the MCID as 0.28 mmHg, corresponding to the minimum detectable effect (MDE) for 80% power with our fixed sample size. Importantly, the entire 95% CI for the observed effect lay below this threshold ($\Delta_{U}=0.15$ mmHg < 0.28 mmHg), yielding a proportion above MCID of $P_{\delta }=0\%$. This means that the range of plausible between-group differences is entirely incompatible with a clinically meaningful benefit of HPI-guided management in reducing hypotension burden.

Thus, while the study was not powered to detect very small differences such as 0.05 mmHg, the confidence interval excludes effects of a magnitude that would be clinically relevant or practice-changing. These findings therefore represent *evidence of absence* of meaningful benefit—rather than merely absence of statistical significance—for the primary endpoint.

### 4.3. Benefits from the HPI Technology in Vascular and Cardiac Surgery

There are contradictory data on the applicability of the HPI technology in major vascular or cardiac surgery. A randomised controlled trial performed in the Netherlands, including 142 patients undergoing elective on-pump coronary artery bypass graft surgery, showed a 63% reduction in median TWA of hypotension with MAP below 65 mmHg with the use of the HPI technology in comparison to the standard care. Patients with HPI haemodynamic monitoring spent a median of almost half an hour less in hypotension, with no significant additional administration of fluids, vasopressors, or inotropes [24].

A study published in 2021 evaluated the applicability of the HPI technology in cardiac surgical patients having cardiopulmonary bypass. The HPI technology appeared to be feasible in this cohort of patients, with sensitivity and specificity at 5 min before the hypotensive episode of 84% and 84%, and at 15 min, 79% and 74%, respectively [25]. Having in mind that cardiopulmonary bypass surgery is frequently accompanied by vasoplegia, affecting almost 50% of cases and being associated with increased mortality (30–50%), the authors analysed the usefulness of the HPI technology before and after CPB. The validity of the HPI was similar before and after CPB. Similarly, surgical manipulations and open vs. closed chest situations did not affect the usefulness of this technology [25,26].

A recently published study showed benefits of the HPI technology in patients undergoing major abdominal aortic surgery [27]. The authors conducted an observational study in 53 patients undergoing open abdominal aortic aneurysm repair with the use of haemodynamic monitoring based on the HPI technology. The study proved that the HPI technology allowed the reduction in the amount of hypotension with MAP of 65 mmHg below 10% of monitoring time, and the TWA for MAP < 65 mmHg was also reduced below the predicted value of 0.40 mmHg [27]. The findings of that study are comparable to those obtained in our cohort, with similar TWA values in the HPI and FloTrac groups (0.22 mmHg and 0.26 mmHg, respectively). Furthermore, as the HPI is an arterial pressure–cardiac output–based technology, its value in reducing episodes of hypotension seems to be quite predictable in different types of surgeries, especially after years of implementing goal-directed therapies in the perioperative period.

### 4.4. Lack of Benefits of the HPI Technology in Vascular and Cardiac Surgery

Despite results confirming the usefulness of the HPI technology in the post-bypass vasoplegia, there are still some doubts about the application of the arterial pressure–derived monitoring techniques in cardiac surgery due to valvular disease, epicardial pacing, or intra-aortic balloon pumps, which all can make the arterial pressure signal unreliable [28].

In the study published in 2019, evaluating the applicability of the HPI in the cardiac and vascular surgery patients, the authors showed that HPI values below 85 carried an acceptable NPV for hypotension and could be utilised by the anaesthesiologist as a “safe zone”. However, values above 85 did not pose a clinically significant PPV to trigger an intervention in order to avoid episodes of hypotension. The authors suggested that higher values of HPI > 98% would be more clinically relevant in this group of patients. [29] However, the small PPV for these approaches might be in line with the ESA/ESAIC guidelines, which recommend avoidance of drops in MAP of more than 20% from baseline or 60–70 mmHg lasting longer than 10 min [30], giving the clinicians time to analyse and provide adequate management.

The results of our retrospective study might be in line with the above-mentioned conclusions, showing that the HPI value of 85 is not useful in vascular surgery, as the clinical interventions applied did not reduce the number of hypotension episodes in comparison to the standard cardiac output monitoring, and on the other hand, resulted in a higher number of hypertension episodes.

### 4.5. HPI Predictive Limitations and MAP-Based Alternatives

There are also concerns regarding the selection bias during the validation of the HPI technology, both in the study by Hatib and most of the successive studies aiming to validate the HPI technology [28,31,32]. The doubts regarding the issue of validation of the HPI technology are related to the fact of the high correlation between the HPI values and mean arterial pressure [33]. This phenomenon is referred to as the mirror effect [34]. The Hypotension Prediction Index value of 85, being the threshold for intervention, corresponds to a mean arterial pressure of 72–76 mmHg. Application of these MAP values as a trigger for an intervention to avoid an episode of hypotension might be as effective as the use of the HPI algorithm [35,36]. There are MAP-based algorithms that predict intraoperative hypotension as well as the HPI technology with a higher MAP threshold for intervention, ranging between 70 and 75 mmHg, making it also a proactive management [37,38].

An observational study published by Mulder in 2024 compared the haemodynamic management based on the HPI alarms and MAP-based alarms. The authors proved almost equal agreement between the two types of alarms to predict an episode of hypotension five minutes before its occurrence. In clinical practice, the HPI triggers an alarm which is almost identical to the alarms triggered by a drop in mean arterial pressure. The results of this study suggest that the alarm generated by the HPI algorithm could be replaced with an alarm based on a mean arterial pressure threshold of 72 or 73 mmHg [37]. Rellum et al. published a study comparing HPI with various MAP thresholds for the prediction of intraoperative hypotension. Surprisingly, the authors concluded that an HPI alert of >85 lasting >40 s is a better predictor of the hypotension than an MAP less than 72 mmHg threshold, without a >40 s alert with a positive predictive value of 55% versus 30%, respectively. Furthermore, they demonstrated a marginally increased time to event of 0.58 min with the utilisation of the HPI algorithm. However, this difference seems to be of no clinical relevance, and the rate of missed hypotensive events was comparable between the HPI and MAP thresholds. Further analysis revealed that comparison of HPI 85 against MAP less than 72 mmHg was similar regarding the positive predictive value of 30% for hypotension. The predictive performance of both of these values was also comparable with the >40 s alert approach, which is clinically reasonable due to the avoidance of alarms caused by short fluctuations in MAP [38,39]. Regarding the additional cost related to the use of the Acumen transducer, the similarity in the rate of hypotension between the HPI technology and simple MAP-based management might have a huge impact on the haemodynamic perioperative standards. There are some randomised clinical trials ongoing which might help in answering this question [40,41].

### 4.6. Unnecessary Interventions Related to HPI

Another domain is the possible rate of false positive alarms and interventions due to HPI or MAP below 72 mmHg alarms, related to the low positive predictive value of both these parameters. Arguments against the use of HPI technology include the possibility of performing unnecessary interventions which might result in worse outcomes [42,43]. This phenomenon can result in overtreatment with higher use of fluids and vasopressors and a higher rate of hypertension episodes, which was clearly shown in our study. The patients in the HPI group have a statistically significant higher number of hypertension episodes with MAP > 90 and 100 mmHg (% of total monitored time). In a prospective randomised study by Wu et al. the authors showed in a group of six hundred seventy-eight elderly patients with chronic hypertension undergoing major gastrointestinal surgery that both hypotensive episodes with MAP below 80 mmHg and hypertensive events with MAP > 96 mmHg were related to the highest risk of acute kidney injury in the postoperative period [44]. Two studies evaluating the HPI technology showed an increased rate of hypertension [45,46]. Another study revealed that haemodynamic management with the use of the Hypotension Prediction Index technology was related to an increased intraoperative blood loss [47]. The data showing a possible relationship between the HPI technology and hypertension might be of particular concern in patients undergoing cardiac surgery. Regarding the reported low predictive value of the HPI in this patient population, applying this algorithm might result in significant overtreatment [28]. It is worth mentioning that not all studies focus on the problem of overtreatment with the use of the HPI technology and do not give data on episodes of hypertension, like, for example, lately published study on the use of the HPI technology in robotic surgery [48]. There are, of course, studies which evaluate the issue of hypertension related to the use of the HPI, which showed there was no difference in the rate of hypertension between the HPI and control groups [46]. On the other hand, in a study by Tsoumpa et al evaluating the HPI algorithm in moderate- and high-risk patients undergoing non-cardiac surgery, the authors found a higher incidence of hypertension with MAP > 100 mmHg in the HPI group. The higher rate of hypertension was not related to an increased amount of crystalloids, colloids, or ephedrine; there was a higher adjusted-to-body-weight use of phenylephrine in the HPI group [45]. Frassanito et al. in a study performed on 70 patients undergoing major gynaecological oncological procedures, also showed a higher incidence of episodes of hypertension with MAP > 110 mmHg. Again, there was no difference in the amount of fluids, vasopressors and transfusion of blood products between the HPI and control groups. The only difference was the higher use of dobutamine in the HPI group. However, the overall incidence of hypertension episodes was low, and the authors of both mentioned studies did not report any harm related to the occurrence of hypertension [49].

The perioperative hypotension debate still lacks answers to some clinically important questions, such as whether better blood pressure control improves perioperative and postoperative outcomes [50,51,52]. A multicentre randomised trial conducted in 28 hospitals and including 917 patients undergoing major elective abdominal surgery compared the rate of moderate and severe AKI during the first seven postoperative days in relation to the type of haemodynamic monitoring. The use of the HPI algorithm in comparison to standard management did not prove any benefit in the rate of acute kidney failure, need for dialysis, overall complications rate, length of hospitalisation and 30-day mortality [50].

Furthermore, results of recently published studies point to the fact that there might be a weaker than previously believed relationship between episodes of hypotension and perioperative complications [42,53,54]. Anaesthesiologists and centres which use the Hypotension Prediction Index algorithm on a daily basis and are familiar with this technology show a low amount of TWA below 65 mmHg, but whether this phenomenon is related to the HPI technology is unknown [35].

### 4.7. Limitations of the Study

The retrospective design of our study introduces inherent limitations that warrant consideration. Retrospective studies are particularly susceptible to selection and information biases, and the absence of randomisation diminishes the robustness of the conclusions.

A critical limitation of this study is the selective nature of the patient cohort. Patients with atrial fibrillation and severe valvular disease were excluded to ensure data quality, as these conditions compromise the reliability of arterial pressure-derived cardiac output monitoring. However, because of the retrospective design, we did not prospectively document the total number of patients screened or the exact number excluded during the study period. As a result, the screening rate and the representativeness of the final cohort relative to the broader population of patients undergoing major aortic vascular surgery at our institution cannot be fully assessed. Therefore, our findings are most applicable to haemodynamically stable vascular surgical patients without significant arrhythmias or structural heart disease, and their generalisability to populations with higher prevalence of such conditions is uncertain. Future prospective studies should systematically track screening and exclusion rates to more accurately characterise generalisability and external validity across diverse vascular surgical populations.

It is also difficult to assess to what extent anaesthesiologists followed the haemodynamic algorithms described in the study and routinely applied in our institution. On the other hand, the retrospective nature of the analysis reflects real-life clinical practice, free from the bias associated with participation in a randomised trial and without the Hawthorne effect.

## 5. Conclusions

In this retrospective study of patients undergoing major vascular aortic surgery, HPI-guided haemodynamic management did not provide clinically meaningful benefit over conventional FloTrac monitoring for reducing hypotension burden. Using a confidence-interval-based framework, the observed effect lay entirely below the threshold for clinical importance, demonstrating equivalence between the two monitoring approaches. However, increased hypertensive exposure with HPI use suggests that algorithm-guided management may require careful calibration to avoid overcorrection. Future prospective randomised controlled trials are needed to determine whether HPI can provide meaningful benefit in broader vascular surgical populations without increasing hypertensive risk.

## Figures and Tables

**Table 1:** Basic demographic, clinical, and perioperative parameters.

Parameter	All	FloTrac	HPI	*p*-Value
Age (years)	69 (65–74)	68 (65–75)	70 (65–73)	0.931
Female, *n* (%)	23 (23%)	13 (26%)	10 (20%)	0.476
BMI	24.6 (24.1–26.5)	24.2 (24.1–26.6)	24.7 (24.2–26.5)	0.577
Weight (kg)	72 (65–82)	70 (65–80.75)	73 (65–83.5)	0.645
ASA III, *n* (%)	–	45 (90%)	45 (90%)	1.000
Hypertension, *n* (%)	82 (82%)	43 (86%)	39 (78%)	0.298
Heart failure, *n* (%)	20 (20%)	5 (10%)	15 (30%)	0.012 *
Coronary artery disease, *n* (%)	37 (37%)	13 (26%)	24 (48%)	0.023 *
Diabetes, *n* (%)	21 (21%)	10 (20%)	11 (22%)	0.806
COPD, *n* (%)	12 (12%)	7 (14%)	5 (10%)	0.538
ACEI, *n* (%)	48 (49.5%)	23 (48.9%)	25 (50%)	0.917
ARB, *n* (%)	4 (4.1%)	3 (6.4%)	1 (2%)	0.352
Betablocker, *n* (%)	54 (55.7%)	30 (63.8%)	24 (48%)	0.117
Calcium-channel blocker, *n* (%)	30 (30.9%)	19 (40.4%)	11 (22%)	0.05
Diuretics, *n* (%)	26 (26.8%)	14 (29.8%)	12 (24%)	0.520
Duration of surgery (min)	144 (112–185)	145 (111–179)	140 (101–185)	0.398
Monitoring time (min)	190 (151–229)	194 (152–230)	178 (150–225)	0.446
Blood loss (mL)	700 (400–1025)	700 (500–1075)	700 (325–1000)	0.860
Blood loss > 1000 mL, *n* (%)	28 (28%)	15 (30%)	13 (26%)	0.656

Note: BMI = body mass index; ASA = American Society of Anesthesiologists physical status classification; COPD = chronic obstructive pulmonary disease. * Statistically significant at p<0.05. No adjustments for multiple comparisons were made for baseline characteristics as these are descriptive.

**Table 2:** Types of vascular surgery in FloTrac and HPI groups.

Type of Vascular Surgery	FloTrac (*n*)	FloTrac (%)	HPI (*n*)	HPI (%)	Total (*n*)	Total (%)
Aorto-Bifemoral Bifurcated Graft	25	50.0	19	38.0	44	44.0
Straight Aortic Graft	10	20.0	19	38.0	29	29.0
Aorto-Bi-Iliac Bifurcated Graft	4	8.0	4	8.0	8	8.0
Graft Replacement	3	6.0	3	6.0	6	6.0
Thoracoabdominal Endovascular Graft (T-branch)	4	8.0	2	4.0	6	6.0
Aorto-Femoral Bypass	1	2.0	0	0.0	1	1.0
Thoracic Endovascular Aortic Repair (TEVAR)	1	2.0	0	0.0	1	1.0
Other	2	4.0	3	6.0	5	5.0

Note: Fisher’s exact test: p=0.4683 (Monte Carlo simulation, 10,000 replicates). No significant differences in procedure type distribution between groups.

**Table 3:** Comparison of vasoactive drugs, crystalloids, total fluids, and blood products — FloTrac vs. HPI. Confidence intervals for median differences are reported as [FloTrac − HPI].

Parameter	FloTrac (Median [IQR])	HPI (Median [IQR])	95% CI (for Difference = FloTrac − HPI)	*p*-Value
Noradrenaline max dose [µg/kg/min]	0.12 (0.10–0.20)	0.15 (0.10–0.24)	[−0.06; 0.00]	0.136
Noradrenaline infusion time [min]	115 (63 – 166)	121.5 (59–155.5)	[−33.00; 30.00]	0.920
Dobutamine max dose [µg/kg/min]	0.00 (0.00–0.00)	0.00 (0.00–5.00)	[−0.00; 0.00]	0.050
Dobutamine infusion time [min]	0.00 (0.00–0.00)	0.00 (0.00–45.00)	[−0.00; 0.00]	0.078
Crystalloids [mL]	1200 (1000–1500)	1500 (1000–1975)	[−500; 0]	0.144
Total fluids [mL]	2000 (1500–2500)	2000 (1500–2500)	[−250; 250]	1.000
RBC transfused [units]	0.00 (0.00–2.00)	0.00 (0.00–2.00)	NA	0.725
FFP transfused [units]	0.00 (0.00–0.00)	0.00 (0.00–2.00)	NA	0.229

Note: RBC = red blood cells; FFP = fresh frozen plasma; CI = confidence interval; IQR = interquartile range. Statistically significant at p<0.05. *p*-values are reported without adjustment for multiple comparisons, as these were not pre-specified study endpoints. Negative CI values indicate that the FloTrac group had lower median values than the HPI group. For example, crystalloid administration showed a median difference in −300 mL (95% CI −500 to 0 mL), indicating a trend toward lower crystalloid use in the FloTrac group, though not reaching statistical significance.

**Table 4:** Parameters related to intraoperative hypotension.

Parameter	All	FloTrac	HPI	*p*-Value	*p*-Value (Holm)
Preoperative MAP	100.5 (91–110)	99.5 (88–115.5)	101.5 (92–109.75)	0.495	0.990
TWA-MAP < 65 mmHg	0.24 (0.14–0.50)	0.26 (0.17–0.52)	0.22 (0.12–0.47)	0.270	0.810
Area under 65 mmHg (mmHg·min)	48.7 (26.6–98.4)	55.3 (33.4–116.9)	38.3 (18.6–83.4)	0.090	0.450
Episodes of hypotension	3.0 (2.0–5.0)	3.5 (2.0–5.0)	3.0 (1.25–4.75)	0.154	0.616
Total hypotension time (min)	8 (3–14)	10 (5–15)	5 (2–11)	0.030 *	0.180
Mean episode duration (min)	2 (2–3)	3 (2–3)	2 (1–3)	0.020 *	0.140
Episodes of MAP < 50 mmHg	0 (0–0.25)	0 (0–0)	0 (0–1)	0.562	0.990

Note: * Statistically significant at p<0.05 before correction; after Holm–Bonferroni correction (n=7 tests) no comparisons remained statistically significant (all $p_{\mathrm{adjusted}}>0.05$).

**Table 5:** Parameters related to intraoperative hypertension.

Parameter	All	FloTrac	HPI	*p*-Value	*p*-Value (Holm)
MAP > 90 mmHg (min)	55 (37–81)	49 (28–81)	62 (49–81)	0.046 *	0.066
MAP > 90 mmHg (% of time)	34 (18–46)	25 (15–40)	38 (26–52)	0.006 *	0.036 **
MAP > 100 mmHg (min)	26 (11–42)	16 (8–36)	31 (18–42)	0.022 *	0.066
MAP > 100 mmHg (% of time)	13 (6–24)	9 (4–20)	18 (9–29)	0.006 *	0.036 **
TWA > 90 mmHg	3.13 (1.92–4.41)	2.42 (1.30–5.23)	4.04 (2.79–7.10)	0.013 *	0.052
TWA > 100 mmHg	1.21 (0.68–1.80)	0.98 (0.31–2.23)	1.61 (0.81–3.02)	0.027 *	0.066

Note: * $p_{\mathrm{uncorrected}}<0.05$; ** $p_{\mathrm{adjusted}}<0.05$ after Holm–Bonferroni correction (*n* = 6 tests).

**Table 6:** Postoperative adverse outcomes and complications comparing HPI and FloTrac groups.

Outcome	FloTrac	HPI	RR (95% CI)	*p*-Value
AKI	19/49 (38.8%)	15/50 (30.0%)	0.77 (0.45–1.34)	0.358
MINS	5/47 (10.6%)	12/49 (24.5%)	2.30 (0.88–6.03)	0.076
Circulatory Failure	9/50 (18.0%)	10/50 (20.0%)	1.11 (0.49–2.50)	0.799
Stroke	0/50 (0.0%)	1/50 (2.0%)	—	1.000
Respiratory Failure	5/50 (10.0%)	4/50 (8.0%)	0.80 (0.23–2.81)	1.000
Reoperation	11/50 (22.0%)	8/50 (16.0%)	0.73 (0.32–1.65)	0.444
Surgical Complications	10/50 (20.0%)	9/50 (18.0%)	0.90 (0.40–2.02)	0.799
Mortality	2/50 (4.0%)	4/50 (8.0%)	2.00 (0.38–10.43)	0.678

Note: AKI—acute kidney injury; MINS—myocardial injury after non-cardiac surgery; RR = relative risk; CI = confidence interval. *p*-values obtained from Fisher’s exact test (with Monte Carlo simulation (10,000 replicates) for outcomes with expected cell frequencies <5, or chi-square test otherwise. No multiple comparison correction applied, as these represent prospectively defined clinically meaningful adverse events rather than exploratory endpoints.

## Data Availability

The datasets generated and/or analysed for this study are currently not publicly available due to their use in other analyses. Selected data, however, are available from the corresponding author upon request.

## Supplementary Materials

The following supporting information can be downloaded at https://www.mdpi.com/article/10.3390/jcm14248791/s1, Table S1: Definitions of postoperative clinical outcomes; Figures S1–S4: Secondary haemodynamic outcome visualisations (TWA MAP > 90 and >100 mmHg, total hypotension time, hypertension duration and percentage).

## Abbreviations

The following abbreviations are used in this manuscript:

HPI	Hypotension prediction index
IOH	Intraoperative hypotension
APCO	Arterial pressure cardiac output
GDT	Goal-directed therapy
MACCE	Major adverse cardiovascular and cerebrovascular events
AKI	Acute kidney injury
MINS	Myocardial injury after non-cardiac surgery
