# Supplementary material for: Hypotension Prediction Index Software Compared with Standard Advanced Haemodynamic Monitoring in Patients Undergoing Major Aortic Surgery: A Retrospective Study
Source: J Clin Med. 2025 Dec 12;14(24):8791. doi: 10.3390/jcm14248791 (PMC12734279; doi:10.3390/jcm14248791)
Supplement: Supplementary file 1 [file jcm-14-08791-s001.zip › Supplement_Figures.pdf]

Supplementary Figures

HPI vs FloTrac Study

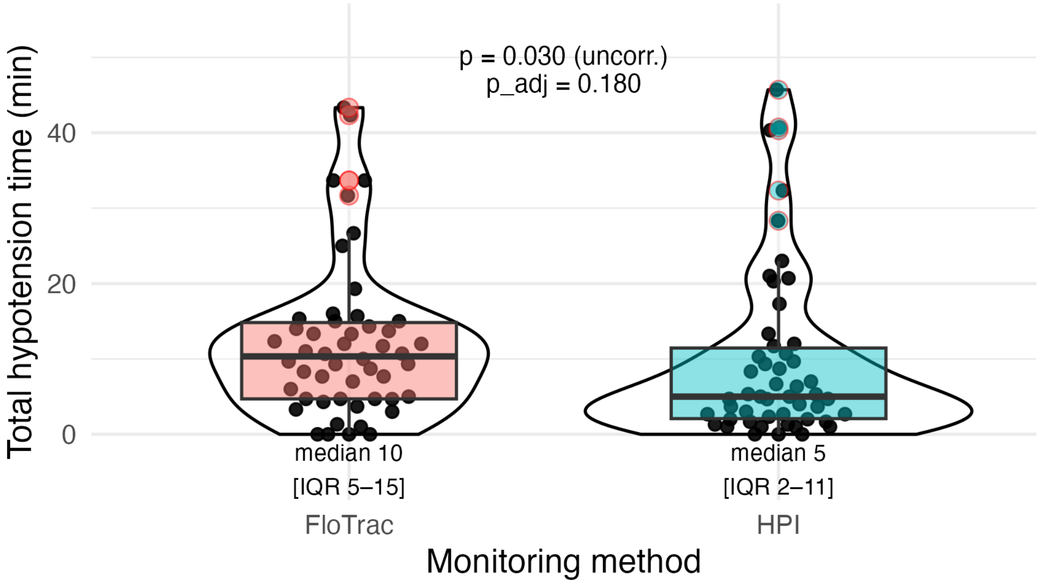

Figure S1. Distribution of total hypotension time (min) in FloTrac vs HPI. Median [IQR]: 10 [5–15] vs 5 [2–11]; $p_{\text{uncorrected}} = 0.030$, $p_{\text{adjusted}} = 0.180$.

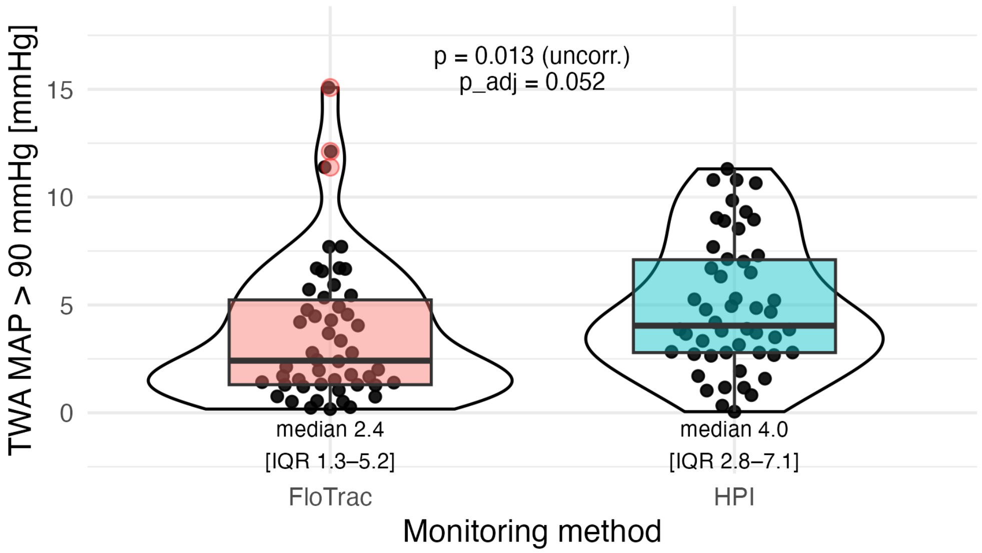

(a) TWA MAP > 90 mmHg

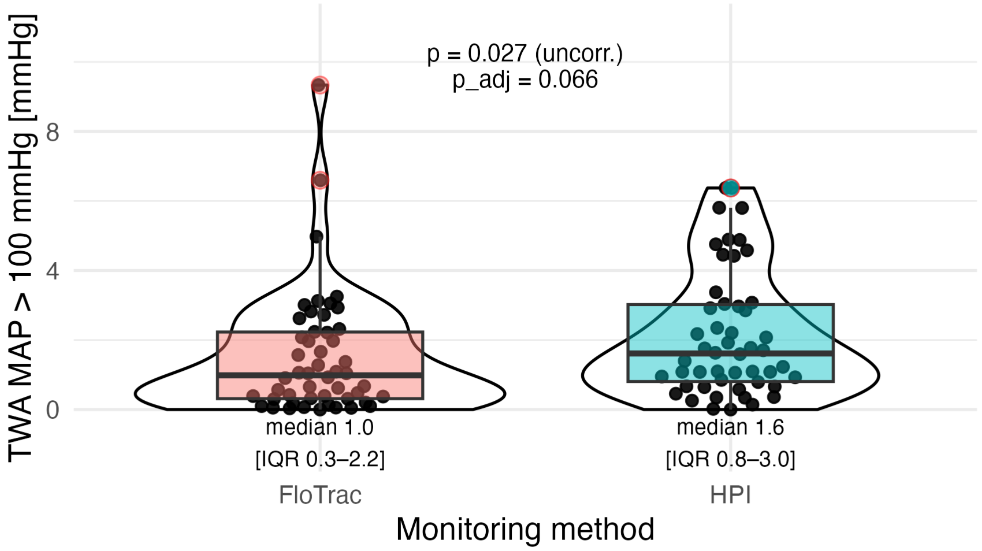

(b) TWA MAP > 100 mmHg

Figure S2. Time-weighted average MAP above predefined thresholds in FloTrac and HPI groups.

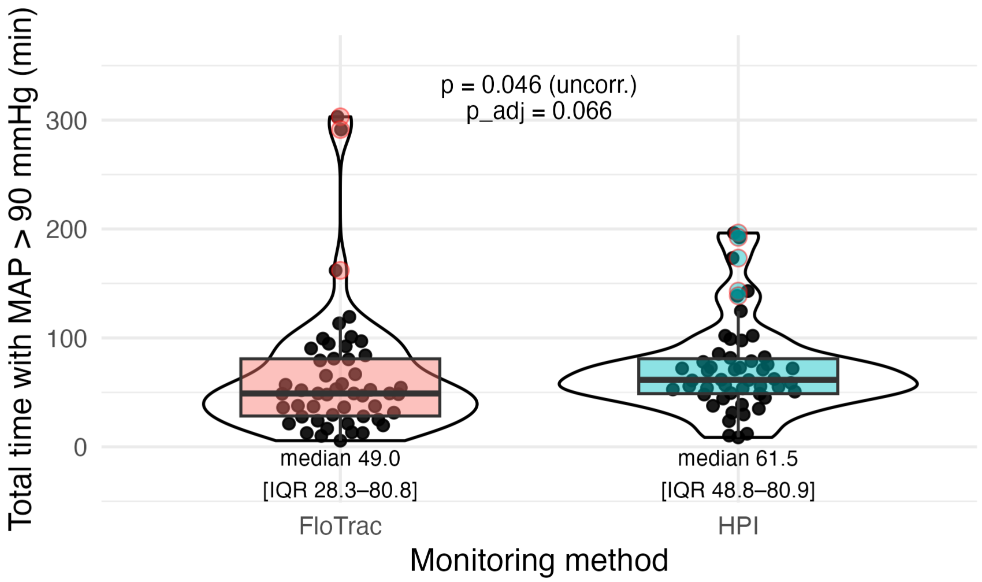

(a) Total time with MAP > 90 mmHg

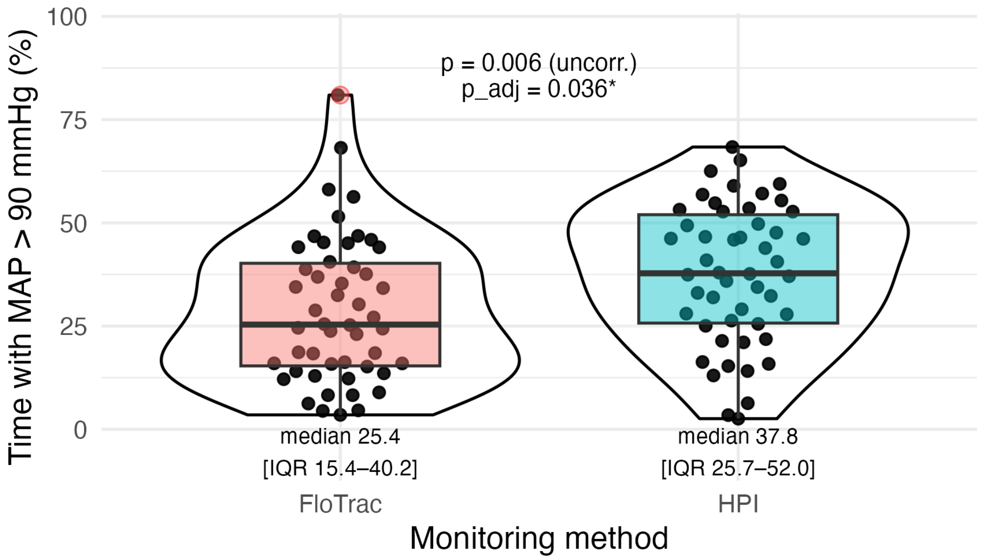

(b) Percentage of monitored time with MAP > 90 mmHg

Figure S3. Hypertension with MAP > 90 mmHg in FloTrac vs HPI groups.

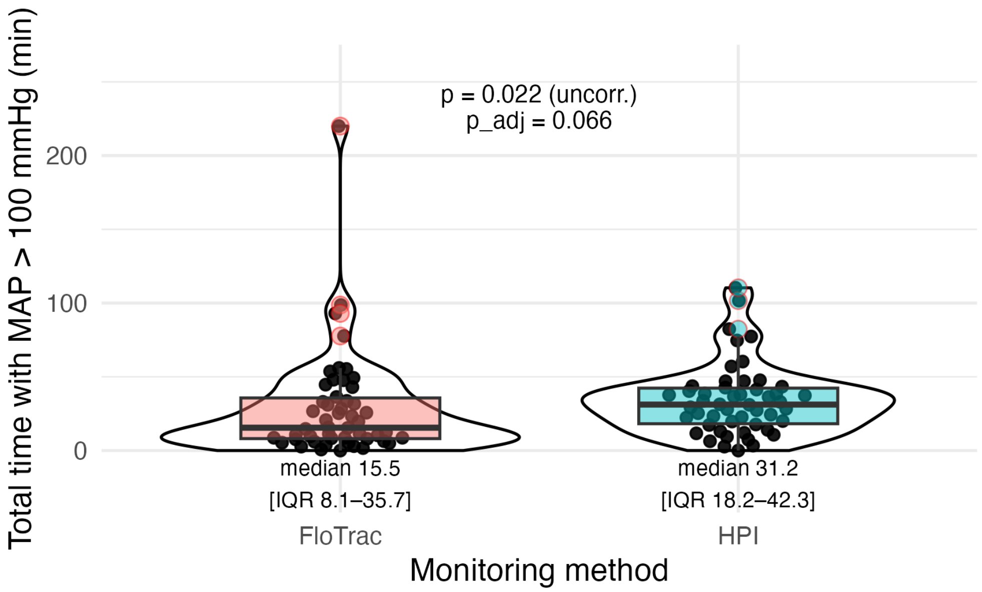

(a) Total time with MAP > 100 mmHg

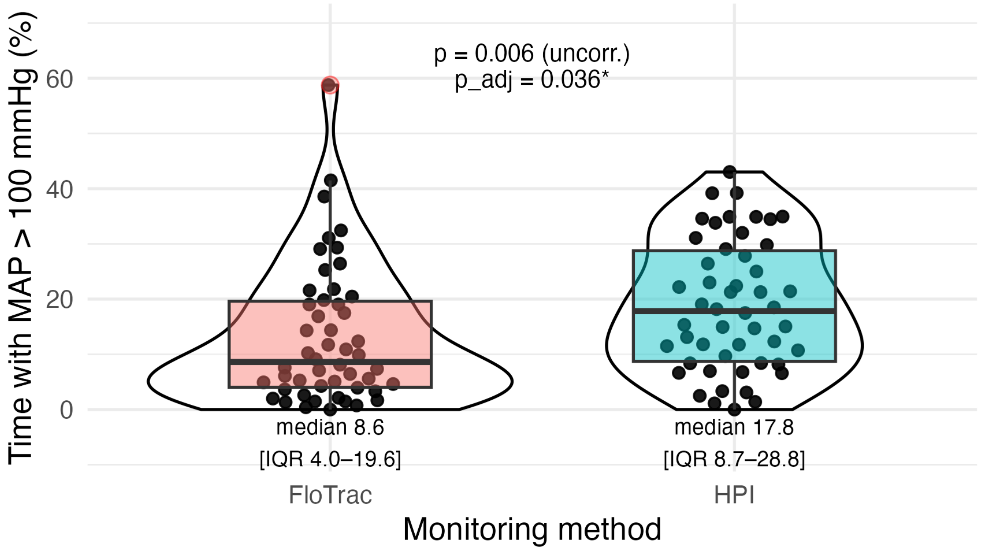

(b) Percentage of monitored time with MAP > 100 mmHg

Figure S4. Hypertension with MAP > 100 mmHg in FloTrac vs HPI groups.
