# Supplementary material for: Hypotension Prediction Index Software Compared with Standard Advanced Haemodynamic Monitoring in Patients Undergoing Major Aortic Surgery: A Retrospective Study
Source: J Clin Med. 2025 Dec 12;14(24):8791. doi: 10.3390/jcm14248791 (PMC12734279; doi:10.3390/jcm14248791)
Supplement: Supplementary file 1 [file jcm-14-08791-s001.zip › Table_S1_OutcomeDefinitions.pdf]

Table S1. Definitions of Postoperative Clinical Outcomes

Outcome	Definition
**Acute Kidney Injury**	Defined according to KDIGO serum creatinine criteria: increase in serum creatinine by $\geq$0.3 mg/dL ($\geq$26.5 µmol/L) within 48 hours or $\geq$1.5 times baseline within 7 days.
**Myocardial Injury After Noncardiac Surgery (MINS)**	At least one postoperative high-sensitivity cardiac troponin value above the assay-specific 99th percentile upper reference limit (URL; 0.034 ng/mL or 0.053 ng/mL depending on platform) within 30 days after surgery, irrespective of the presence of ischaemic symptoms or ECG changes.
**Postoperative Respiratory Failure**	Composite endpoint reflecting clinically relevant impairment of gas exchange or ventilation. Present if at least one criterion was met within 7 days after surgery or before ICU discharge:<br>• Prolonged invasive mechanical ventilation (failure to extubate within 48 hours)<br>• Unplanned reintubation due to respiratory failure after initial extubation<br>• Unplanned noninvasive ventilation or high-flow oxygen maintained for $\geq$6 hours
**Postoperative Circulatory Failure**	Composite endpoint reflecting clinically relevant cardiovascular instability. Present if at least one criterion was met within 48 hours after surgery:<br>• Sustained vasopressor support (norepinephrine $\geq$0.1 µg/kg/min for $\geq$6 hours)<br>• Initiation or escalation of inotropic therapy for tissue hypoperfusion<br>• Need for mechanical circulatory support (IABP, ECMO, or equivalent)
**Stroke**	New postoperative focal neurological deficit confirmed by neuroimaging.
**Surgical Complications**	Clinically significant postoperative events documented during index hospitalisation: bleeding requiring reoperation or transfusion of $\geq$4 units RBC within 24 hours; evisceration; intestinal ischaemia; acute limb ischaemia requiring intervention; anastomotic leak; visceral organ ischaemia (liver, spleen, pancreas, kidneys).
**Reoperation**	Return to the operating theatre for any surgical complication during index hospitalisation.
**In-hospital Mortality**	Death from any cause during index hospitalisation.
